# Phase 1 Pharmacokinetic Study of AZD5718 in Healthy Volunteers: Effects of Coadministration With Rosuvastatin, Formulation and Food on Oral Bioavailability

**DOI:** 10.1002/cpdd.756

**Published:** 2019-12-02

**Authors:** Hans Ericsson, Karin Nelander, Maria Heijer, Magnus Kjaer, Eva‐Lotte Lindstedt, Muna Albayaty, Pablo Forte, Maria Lagerström‐Fermér, Stanko Skrtic

**Affiliations:** ^1^ Clinical Pharmacology, ADME and AI Clinical Pharmacology & Safety Sciences, BioPharmaceuticals R&D, AstraZeneca Gothenburg Sweden; ^2^ Clinical Pharmacology Biologics and Bioanalysis Clinical Pharmacology & Safety Sciences, R&D, AstraZeneca Gothenburg Sweden; ^3^ Early Biometrics and Statistical Innovation, Data Science & AI BioPharmaceuticals R&D, AstraZeneca Gothenburg Sweden; ^4^ Research and Early Development, Cardiovascular Renal and Metabolic, BioPharmaceuticals R&D, AstraZeneca Gothenburg Sweden; ^5^ Parexel Early Phase Clinical Unit Harrow UK; ^6^ Department of Internal Medicine and Clinical Nutrition Institute of Medicine, Sahlgrenska Academy, University of Gothenburg Gothenburg Sweden

**Keywords:** 5‐lipoxygenase‐activating protein, coronary artery disease, drug‐drug interaction, leukotriene, phase 1 clinical trial, statin

## Abstract

AZD5718 is a first‐in‐class small‐molecule anti‐inflammatory drug with the potential to reduce the residual risk of cardiovascular events after myocardial infarction in patients receiving lipid‐lowering statin therapy. Leukotrienes are potent proinflammatory and vasoactive mediators synthesized in leukocytes via 5‐lipoxygenase and 5‐lipoxygenase‐activating protein (FLAP). AZD5718 is a FLAP inhibitor that dose‐dependently reduced leukotriene biosynthesis in a first‐in‐human study. We enrolled 12 healthy men in a randomized, open‐label, crossover, single‐dose phase 1 pharmacokinetic study of AZD5718 to investigate a potential drug‐drug interaction with rosuvastatin, and the effects of formulation and food intake (ClinicalTrials.gov identifier: NCT02963116). Rosuvastatin (10 mg) were absorbed more rapidly when coadministered with AZD5718 (200 mg), probably owing to weak inhibition of hepatic statin uptake, but relative bioavailability was unaffected (geometric least‐squares mean ratio [GMR], 100%; 90% confidence interval [CI], 86%‐116%). AZD5718 pharmacokinetics were unaffected by coadministration of rosuvastatin. AZD5718 (200 mg) was absorbed less rapidly when formulated as tablets than oral suspension, with reduced relative bioavailability (GMR, 72%; 90%CI, 64%‐80%). AZD5718 absorption was slower when 200‐mg tablets were taken after a high‐fat breakfast than after fasting, but relative bioavailability was unaffected (GMR, 96%; 90%CI, 87%‐106%). In post hoc pharmacodynamic simulations, plasma leukotriene B_4_ levels were inhibited by >90% throughout the day following once‐daily AZD5718, regardless of formulation or administration with food. AZD5718 was well tolerated, with no severe or serious adverse events. These data supported the design of a phase 2a efficacy study of AZD5718 in patients with coronary artery disease.

The management of patients with coronary artery disease involves controlling risk factors for disease progression through education and lifestyle modification, as well as pharmacological therapy with antihypertensive, antiplatelet, and lipid‐lowering drugs.^1^ Despite the effectiveness of statins in reducing low‐density lipoprotein (LDL) cholesterol levels, patients remain at risk of acute coronary syndrome events, and cardiovascular disease remains the leading cause of death worldwide.^2^

Inflammation and immune dysregulation are important drivers in the development and progression of cardiovascular disease, independent of high LDL cholesterol levels.^3^ A residual risk of cardiovascular events in statin‐treated patients with coronary artery disease was revealed in a recent phase 3 clinical trial of the proprotein convertase subtilisin/kexin type 9‐neutralizing antibody evolocumab.^4^ Patients receiving evolocumab together with statins had very low LDL cholesterol levels (median, 30 mg/dL) but only a small reduction in the rate of recurrent cardiovascular events compared with those receiving placebo (9.8% vs 11.3%; hazard ratio, 0.85; *P* < .001).^4^ Furthermore, patients’ residual LDL cholesterol‐independent risk can be reduced with anti‐inflammatory therapy, as recently demonstrated in the Canakinumab Anti‐inflammatory Thrombosis Outcome Study (CANTOS).^5^ Patients receiving the anti‐interleukin‐1β antibody canakinumab together with statins had a significantly lower rate of recurrent cardiovascular events than those receiving placebo and statins, independent of lipid‐level‐lowering (3.90 vs 4.50 events per 100 person‐years; hazard ratio, 0.85; *P* = .021).

CANTOS investigated interleukin‐1β inhibition, but several other inflammatory pathways are involved in cardiovascular disease, including those mediated by leukotrienes.^3^ Leukotrienes are potent proinflammatory and vasoactive mediators that are produced in leukocytes from arachidonic acid.^6^ Leukotrienes play important roles in atherosclerotic plaque progression and myocardial ischemia in patients with acute coronary syndrome.^7^, ^8^, ^9^, ^10^

The first step in leukotriene biosynthesis is the formation of the unstable precursor leukotriene A_4_ by 5‐lipoxygenase in combination with 5‐lipoxygenase‐activating protein (FLAP). Multiple lines of evidence indicate that drugs targeting FLAP or 5‐lipoxygenase to inhibit leukotriene production in coronary circulation could reduce mortality and prevent acute coronary syndrome in patients with coronary artery disease. First, vasoconstrictive responses to leukotrienes C_4_ and D_4_ are enhanced in coronary artery ring segments from patients with atherosclerosis.^11^ Second, certain haplotypes of the gene encoding FLAP (*ALOX5AP*) are associated with increased risk of myocardial infarction.^12^ Third, the 5‐lipoxygenase inhibitor VIA‐2291 reduced leukotriene B_4_ production, lowered noncalcified plaque volume, and prevented the appearance of new coronary lesions in patients with acute coronary syndrome in a phase 2 clinical trial.^13^ VIA‐2291 also dose‐dependently improved left ventricular ejection fraction in patients with acute coronary syndrome.^13^ Finally, the 5‐lipoxygenase inhibitor zileuton reduced leukotriene production and improved flow‐mediated dilation in the brachial artery in patients with coronary artery disease.^14^

AZD5718 (Figure 1) is a novel and potent FLAP inhibitor that blocks leukotriene B_4_ production in human leukocytes, with a half‐maximal inhibitory concentration (IC_50_) of 39 nM in vitro.^15^ A first‐in‐human phase 1 study of AZD5718 in healthy volunteers showed that single oral doses of 25‐1200 mg and multiple doses of 60‐600 mg were well tolerated, with no identified safety concerns (NCT02632526).^15^ Following administration of an oral suspension, AZD5718 was rapidly absorbed and had a terminal half‐life of 10‐12 hours in plasma, with steady‐state levels reached after about 3 days.^15^ AZD5718 dose‐dependently inhibited leukotriene B_4_ production, as assessed using a whole‐blood ex vivo stimulation assay, with an IC_50_ of 5.3 nM (95% confidence interval [CI], 4.8‐5.8 nM). AZD5718 also dose‐dependently reduced endogenous leukotriene E_4_ levels in urine, with an IC_50_ of 0.8 nM (95%CI, 0.7‐1.0 nM).

**Figure 1:**
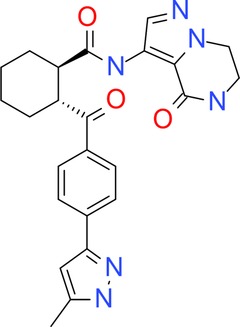
Structure of AZD5718 ((1*R*,2*R*)‐2‐{4‐[3‐Methyl‐1‐(tetrahydro‐2*H*‐pyran‐2‐yl)‐1*H*‐pyrazol‐5‐yl]benzoyl}‐N‐(4‐oxo‐4,5,6,7‐tetrahydropyrazolo[1,5‐*a*]pyrazin‐3‐yl)cyclohexanecarboxamide).^16^

In vitro studies indicate that cytochrome P450 (CYP) 3A4 and CYP3A5 are involved in the metabolism of AZD5718, with glucuronidation by uridine 5’‐diphosphate glucuronosyltransferases 1A1, 1A3, and 1A4 also contributing to metabolism.^15^, ^16^ AZD5718 does not inhibit CYP isoforms 1A2, 2C8, 2C9, 2C19, 2D6, or 3A4 (IC_50_ > 20 µM)^16^ or P‐glycoprotein (IC_50_ > 100 µmol/L). Together with the almost complete inhibition of leukotriene B_4_ and E_4_ production in healthy volunteers in the low nanomolar range, in vitro profiling of AZD5718 indicates low risk of drug‐drug interactions with AZD5718 via CYP450 inhibition or induction or at the transporter level. However, unpublished in vitro studies indicated that AZD5718 is a weak inhibitor of the hepatic statin transporters organic anion‐transporting polypeptide 1B1 (OATP1B1; IC_50 _ = 2.3 µM) and breast cancer resistance protein (BCRP; IC_50_ = 20 µM).^17^

Here, we present results from a second phase 1 study of AZD5718 in healthy volunteers (NCT02963116). The study aimed to investigate a potential drug‐drug interaction between AZD5718 and rosuvastatin in vivo.^18^ High‐dose statin therapy is indicated in the AZD5718 target population, making early quantitative understanding of any potential interaction important. We also investigated the pharmacokinetics of AZD5718 when administered as an immediate‐release tablet compared with an oral suspension and when taken in the fed or fasted state. AZD5718 pharmacodynamics under these conditions were predicted using post hoc simulations of plasma leukotriene B_4_ levels, because leukotriene levels were not measured in the study. These results supported the design and dosing regimen of a recently initiated phase 2a study investigating the efficacy, safety, and tolerability of AZD5718 oral tablets in patients with coronary artery disease (NCT03317002; Eva Prescott et al, in preparation).

## Methods

### Overview and Objectives

This was a randomized, open‐label, 5‐period, 5‐treatment, crossover phase 1 study of single oral doses of AZD5718 in healthy adults (ClinicalTrials.gov identifier: NCT02963116). The primary objective was to evaluate the pharmacokinetics of oral rosuvastatin when administered alone and in combination with oral AZD5718. The secondary objectives were to assess the relative bioavailability of AZD5718 formulated as an immediate‐release tablet compared with an oral suspension; to examine the pharmacokinetic profiles of AZD5718 formulated as an immediate‐release tablet when administered in fed and fasted conditions, and to assess the safety and tolerability of single doses of AZD5718 in healthy adults.

Leukotriene B_4_ levels were not measured in the present study, limiting the ability to draw conclusions on the potential therapeutic dose for the immediate‐release tablet formulation. Instead, post hoc pharmacodynamic simulations were performed using the observed pharmacokinetic profiles of AZD5718 to predict the effect of multiple doses of AZD5718 on plasma leukotriene B_4_ levels. These simulations relied on a pharmacokinetic‐pharmacodynamic model, which was developed based on results from the first‐in‐human phase 1 study.^15^ This approach allowed the results of the present study to support the design of the currently ongoing phase 2a study of AZD5718 (NCT03317002).

### Conduct and Ethics

The study took place between December 2016 and March 2017 at the Parexel Early Phase Clinical Unit in Northwick Park Hospital, London, UK. It was conducted in accordance with the principles of the Declaration of Helsinki and the International Conference on Harmonisation Good Clinical Practice. An independent Ethics Committee at the study site (South Central, Berkshire B Research Ethics Committee) and the Medicines and Healthcare Products Regulatory Authority reviewed and approved the study protocol and its amendments. All participants freely gave their written informed consent before entering the study. The study was registered with ClinicalTrials.gov (identifier: NCT02963116).

### Study Design

Each participant received 5 single‐dose oral study treatments (A‐E), with a minimum washout period of 7 days between each treatment period. Participants were randomly assigned to treatments in the order A, B, C, D, E or B, A, C, D, E. Treatments were: A, rosuvastatin 10‐mg tablet; B, rosuvastatin 10‐mg tablet plus AZD5718 200‐mg immediate‐release tablet; C, AZD5718 200‐mg immediate‐release tablet; D, AZD5718 200‐mg oral suspension; and E, AZD5718 200‐mg immediate‐release tablet administered after food (treatments A‐D were administered after fasting).

Participants fasted for at least 10 hours before treatment and for at least 4 hours after treatment. Participants receiving treatment E consumed a high‐fat breakfast 30 minutes before treatment. Water was permitted as desired except within 1 hour of administration. The AZD5718 200‐mg immediate‐release tablet was given as two 100‐mg tablets, and the AZD5718 oral suspension was 50 mg/mL. Doses were taken with 240 mL of water.

During each treatment period, participants resided at the study center from the morning before treatment (day ‐1) until at least 48 hours after dosing (day 3). Participants attended a follow‐up visit 7‐10 days after their final dose in the study.

### Participants

Eligible participants were healthy men and women aged 18‐50 years weighing 50‐100 kg with a body mass index of 18‐30 kg/m^2^. Key exclusion criteria were history or presence of any disease or disorder that might influence study participation or results; history or presence of any condition known to interfere with absorption, distribution, metabolism, or excretion of drugs; any clinically significant illness, medical procedure, or trauma within the previous 4 weeks; any clinically important abnormalities in hematology, urinalysis, or blood chemistry (including aminotransferase, aspartate aminotransferase, total bilirubin, or gamma‐glutamyl transferase levels above the upper limit of normal); abnormal vital signs (including systolic blood pressure < 90 or ≥ 140 mm Hg, diastolic blood pressure < 50 or ≥ 90 mm Hg, and pulse < 45 or > 85 beats per minute); and any clinically important electrocardiographic abnormalities. Women who were lactating or of childbearing potential were excluded.

### Bioanalytical Methods

Samples for determination of AZD5718 in plasma were analyzed by Covance Laboratories (Harrogate, UK), and samples for determination of rosuvastatin in plasma were analyzed by Covance (Madison, Wisconsin).^15^, ^19^ The bioanalytical methods were validated prior to sample analysis, and all study samples were analyzed within the known stability period. At a minimum, each analytical run included a calibration curve, a matrix blank, a control zero sample (matrix blank containing internal standard), a reagent blank, and duplicate quality control samples at 3 concentrations within the calibration range. Both methods also demonstrated selectivity in the presence of coadministered drug. To demonstrate acceptable in‐study performance, incurred sample reproducibility analyses were performed during the study. For determination of AZD5718 in plasma, 73 of the 74 samples (98.6%) tested were within 20% of the mean of the 2 values. For determination of rosuvastatin, 34 of the 36 samples (94.4%) tested were within 20% of the mean of the 2 values. Both assessments were well within the acceptance criteria.

### Pharmacokinetic Analyses

Plasma samples were collected for pharmacokinetic analyses before dosing and 0.5, 1, 2, 3, 4, 5, 6, 8, 10, 12, 18, 24, 36, and 48 hours after dosing with each treatment. Plasma pharmacokinetic end points for rosuvastatin were area under the plasma concentration‐time curve from time 0 to infinity (AUC_0‐∞_), area under the plasma concentration‐time curve from time 0 to time of last quantifiable concentration (AUC_0‐last_), maximum observed plasma concentration (C_max_), time to reach maximum observed plasma concentration (t_max_), and half‐life associated with terminal slope of a semilogarithmic concentration‐time curve (t_½λz_). Pharmacokinetic end points for AZD5718 were AUC_0‐∞_, AUC_0‐last_, C_max_, t_max_, t_½λz_, and total body clearance of drug from plasma (CL/F).

The pharmacokinetic analysis set for rosuvastatin included all participants who received both rosuvastatin treatments. The pharmacokinetic analysis set for AZD5718 included all participants who received at least 1 AZD5718 treatment. Both analysis sets included only participants with at least 1 calculable pharmacokinetic end point and without major protocol deviations that could affect the pharmacokinetic analyses. Pharmacokinetic parameters for AZD5718 were expressed in molar units (molecular weight, 446.5 g/mol).

### Sample Size Calculation

The number of participants in the study was selected to enable collection of a sufficient amount of data on the primary end points while exposing as few volunteers as possible to study procedures. Assuming a true intrasubject coefficient of variation (CV) of 20% for area under the curve (AUC), a sample size of 10 evaluable participants was estimated to provide a relative precision of 1.5 (ratio between the upper and lower limits of the 90%CI) with 80% probability. This corresponds to a 90%CI of 1.63‐2.45 assuming a treatment ratio of 2.0. To account for potential discontinuations, 12 subjects were included in this study.

### Statistical Analyses

Statistical analyses were performed by Parexel using SAS version 9.4. Rosuvastatin pharmacokinetics were analyzed using a mixed‐effects model, with log‐transformed AUC, AUC_0‐last_, and C_max_ as the response variables; treatment sequence, period, and treatment as fixed effects; and participant nested within treatment sequence as a random effect. Least‐squares (LS) mean differences and 90%CIs for administration alone versus coadministration with AZD5718 were back‐transformed to geometric mean ratios and presented as percentages with corresponding 90%CIs. AZD5718 pharmacokinetics were modeled using an analysis of variance of log‐transformed AUC, AUC_0‐last_, and C_max_, with either formulation or food as a fixed effect and participant as a random effect. LS mean differences and 90%CIs for the effect of formulation or food for log‐transformed variables were back transformed to geometric mean ratios and presented as percentages with 90% CIs.

### Post Hoc Pharmacokinetic‐Pharmacodynamic Analyses

Pharmacodynamic simulations were performed using the observed pharmacokinetic profiles of AZD5718 to predict plasma leukotriene B_4_ levels after multiple doses. These simulations relied on a population pharmacokinetic‐pharmacodynamic model developed based on data from the first‐in‐human phase 1 study of AZD5718.^15^

The 2‐compartment structural pharmacokinetic model assumed first‐order absorption of AZD5718 and linear elimination from the central compartment. Nonlinearity in the pharmacokinetic relationship between bioavailability and dose was modeled as a power law, scaled to AZD5718 180 mg. Between‐participant variability was estimated for relative bioavailability and the volume of the central compartment. AZD5718 concentration was log‐transformed, and residual error was additive on the log scale. The pharmacodynamic model was added sequentially to the pharmacokinetic model: the pharmacokinetic parameters were first fitted using the pharmacokinetic model, then fixed in the pharmacodynamic model. The pharmacodynamic model used a peripheral effect compartment with sigmoidal dependence of leukotriene B_4_ inhibition on drug concentration, according to the following equation:

$$\left[Leukotriene\;B_{4}\right]\;=E_{0}\;-E_{0}\;.\;\frac{C_{E}^{\mathrm{hill}}}{\left(C_{E}^{\mathrm{hill}}+{\;\mathrm{IC}}_{50}^{\mathrm{hill}}\right)}$$

where *E_0_* is the leukotriene B_4_ level at baseline, *C_E_* is the concentration in the effect compartment, *IC_50_* is the half‐maximal inhibitory concentration in the effect compartment, and *hill* is the curve‐fitting parameter. Between‐participant variability was estimated for IC_50_. Leukotriene B_4_ concentration was log‐transformed, and residual error was additive on the log scale.

Pharmacodynamic simulations were used to compare changes in leukotriene B_4_ levels from baseline after multiple doses of AZD5718 administered as suspension or as a tablet and after fasting or after food, based on single‐dose pharmacokinetic data. The pharmacokinetic model was reestimated using combined concentration data from the first‐in‐human phase 1 study^15^ and the 4 AZD5718 treatments (B‐E) from the present study. Pharmacokinetic parameters for individual participants receiving different treatments in the present study were first predicted using the above pharmacokinetic model, then used as inputs in pharmacodynamic simulations of leukotriene B_4_ levels in plasma, based on the above pharmacodynamic model. Baseline leukotriene B_4_ levels were set to 100 to model percentage changes.

Pharmacokinetic‐pharmacodynamic modeling was conducted in accordance with guidelines from the Food and Drug Administration (1999) and the European Medicines Agency (the Committee for Medicinal Products for Human Use, 2007), and using NONMEM 7.3 (Icon Development Solutions, Ellicott City, Maryland). Pharmacodynamic simulations were conducted using the mrgsolve package in R (Foundation for Statistical Computing, Vienna, Austria).

### Safety and Tolerability Outcomes

The safety set included all participants who received treatment in the study and for whom any postdose safety data were available. Safety was assessed throughout the study by monitoring adverse events, vital signs (systolic and diastolic blood pressure, pulse rate, and body temperature), and electrocardiographic parameters and by conducting physical examinations and laboratory assessments (hematology, blood chemistry, and urinalysis).

## Results

### Participants

Twelve healthy men aged 21‐46 years were enrolled and randomized (Table 1). No women met the inclusion criteria. All 12 participants received treatment, completed the study, and were included in the safety and pharmacokinetic analysis sets.

**Table 1:** Participant Demographics

Variable	All Participants (n = 12)
Age, years	
Mean (SD)	34.7 (8.0)
Median (range)	35.0 (21‐46)
Race, n (%)	
White	10 (83.3)
Asian	2 (16.7)
Height, cm	
Mean (SD)	180.5 (6.2)
Median (range)	181.5 (171‐191)
Weight, kg	
Mean (SD)	74.81 (11.01)
Median (range)	71.30 (61.0‐95.1)
BMI, kg/m^2^	
Mean (SD)	22.92 (2.71)
Median (range)	22.45 (18.8‐28.1)

BMI, body mass index; SD, standard deviation.

### Pharmacokinetics and Simulated Pharmacodynamics

#### Coadministration of Rosuvastatin With AZD5718

**Figure 2:**
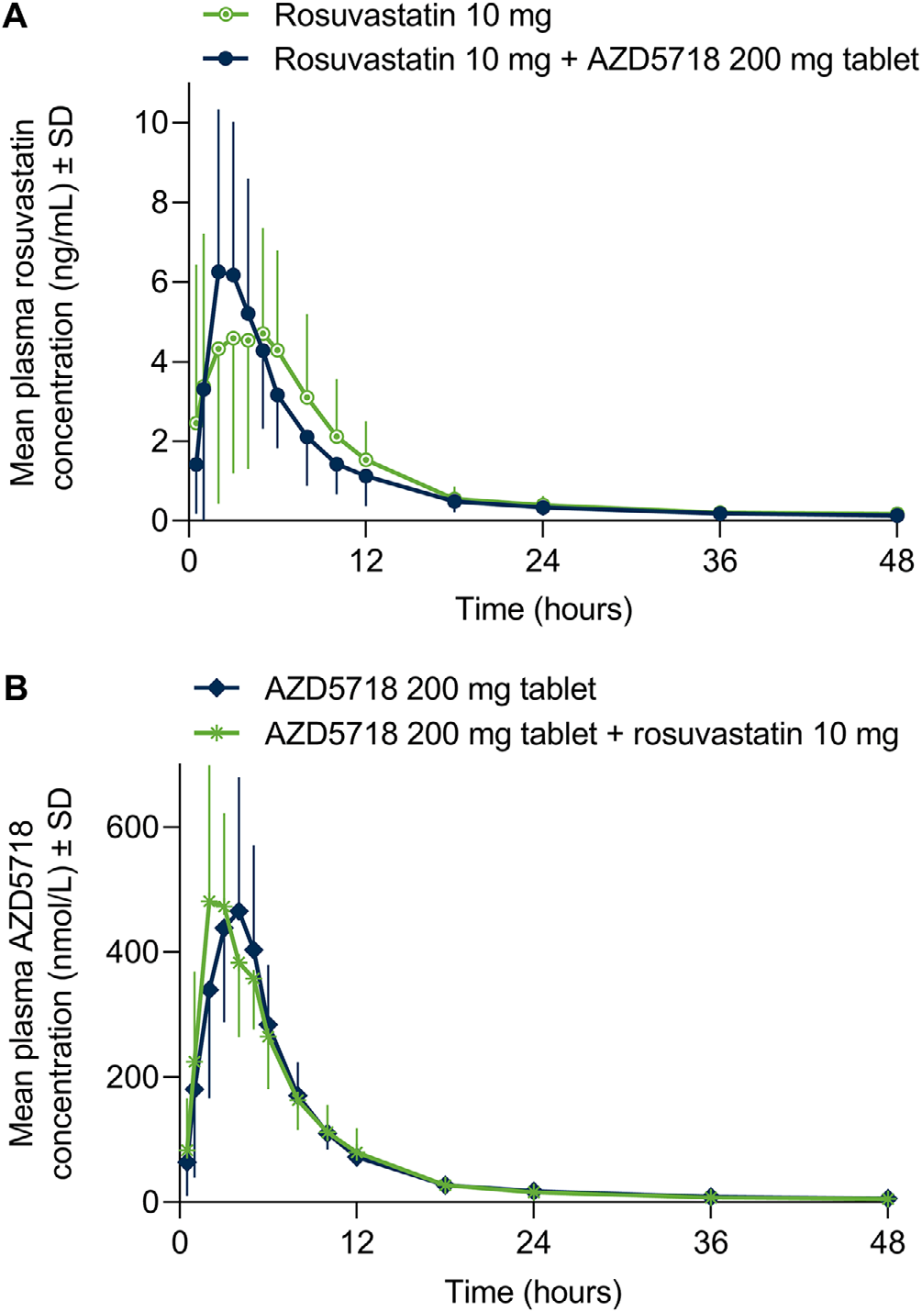
Single‐dose plasma concentration–time profiles of rosuvastatin (A) and AZD5718 (B) after administration alone or coadministration. Molecular weight of AZD5718 is 446.5 g/mol.

Systemic exposure to rosuvastatin was similar when administered alone and when coadministered with AZD5718 (Figure 2A; Table 2). Relative to administration alone, the AUC_0‐∞_ of rosuvastatin coadministered with AZD5718 was 100% (90%CI, 86%‐116%), expressed as a ratio of geometric LS means (and AUC_0‐last_ was 96% [90%CI, 87%‐106%]). Rosuvastatin was absorbed more rapidly when coadministered with AZD5718 than when given alone, with increased C_max_ and decreased t_max_ (Figure 2A; Table 2). The C_max_ of rosuvastatin increased to 125% (90%CI, 107%‐146%) of the value for administration alone, expressed as a ratio of geometric LS means. Coadministration with AZD5718 had little or no effect on the elimination half‐life of rosuvastatin (Table 2). The pharmacokinetics of AZD5718 were not affected by coadministration with rosuvastatin (Figure 2B; Table 3).

**Table 2:** Rosuvastatin Pharmacokinetic Parameters

	Fasted	
Parameter	Rosuvastatin 10 mg (n = 12)	Rosuvastatin 10 mg Plus AZD5718 200‐mg Tablet (m = 12)
AUC_0‐∞_, ng·h/mL		
Arithmetic mean (SD)	45.30 (21.87)^a^	56.91 (30.41)^b^
Geometric mean (%CV)	41.00 (50.3)^a^	50.47 (54.9)^b^
AUC_0‐last_, ng·h/mL		
Arithmetic mean (SD)	54.19 (36.22)	49.39 (28.28)
Geometric mean (%CV)	45.26 (67.6)	43.46 (54.7)
C_max_, ng/mL		
Arithmetic mean (SD)	5.560 (3.773)	6.738 (4.219)
Geometric mean (%CV)	4.590 (72.1)	5.743 (62.9)
t_max_, h		
Median (range)	5.00 (0.50‐6.00)	2.00 (2.00‐5.00)
t_½λz_, h		
Arithmetic mean (SD)	16.0 (6.09)^a^	16.4 (5.72)^b^
Geometric mean (%CV)	14.7 (39.6)^a^	16.5 (29.5)^b^

AUC_0‐∞_, area under the plasma concentration‐time curve from time 0 to infinity; AUC_0‐last_, area under the plasma concentration‐time curve from time 0 to time of last quantifiable concentration; C_max_, maximum observed plasma concentration; CV, coefficient of variation; SD, standard deviation; t_max_, time to reach maximum observed plasma concentration; t_½λz_, plasma half‐life associated with terminal slope of a semilogarithmic concentration‐time curve.

a n = 9.

b n = 10.

**Table 3:** AZD5718 Pharmacokinetic Parameters

	AZD5718 200 mg			
	Fasted			Fed
Parameter	Tablet Plus Rosuvastatin 10 mg (n = 12)	Tablet (n = 12)	Suspension (n = 12)	Tablet (n = 12)
AUC_0‐∞_, nmol·h/L				
Arithmetic mean (SD)	3674 (824.0)	3623 (710.4)^a^	5000 (1102)^a^	3397 (748.4)
Geometric mean (%CV)	3591 (22.6)	3559 (20.1)^a^	4892 (22.2)^a^	3330 (20.5)
AUC_0‐last_, nmol·h/L				
Arithmetic mean (SD)	3548 (832.9)	3491 (667.5)	4751 (1046)	3268 (699.5)
Geometric mean (%CV)	3459 (24.0)	3432 (19.7)	4649 (21.8)	3207 (19.9)
C_max_, nmol/L				
Artihmetic mean (SD)	567.1 (165.3)	565.3 (191.2)	1246 (485.1)	426.8 (147.7)
Geometric mean (%CV)	544.9 (30.4)	530.2 (41.1)	1170 (37.3)	409.2 (29.0)
t_½λz_, h				
Arithmetic mean (SD)	15.0 (7.24)	14.0 (5.35)^a^	13.6 (3.36)^a^	13.7 (2.46)
Geometric mean (%CV)	13.9 (38.4)	13.2 (34.4)^a^	13.2 (25.2)^a^	13.5 (18.4)
t_max_, h				
Median (range)	2.01 (2.00‐5.05)	3.00 (2.00‐5.00)	1.00 (0.98‐2.02)	4.00 (3.00‐5.98)
CL/F, L/h				
Arithmetic mean (SD)	127.6 (28.12)	128.1 (25.63)^a^	93.55 (20.40)^a^	136.9 (25.53)
Geometric mean (%CV)	124.6 (22.6)	126.0 (20.1)^a^	91.5 (22.2)^a^	134.4 (20.4)

AUC_0‐∞_, area under the plasma concentration‐time curve from time 0 to infinity; AUC_0‐last_, area under the plasma concentration‐time curve from time 0 to time of last quantifiable concentration; C_max_, maximum observed plasma concentration; CL/F, total body clearance of drug from plasma; CV, coefficient of variation; SD, standard deviation; t_max_, time to reach maximum observed plasma concentration; t_½λz_, plasma half‐life associated with terminal slope of a semilogarithmic concentration‐time curve.

Molecular weight of AZD5718 is 446.5 g/mol.

a n = 10.

### AZD5718 Immediate‐Release Tablet or Oral Suspension

**Figure 3:**
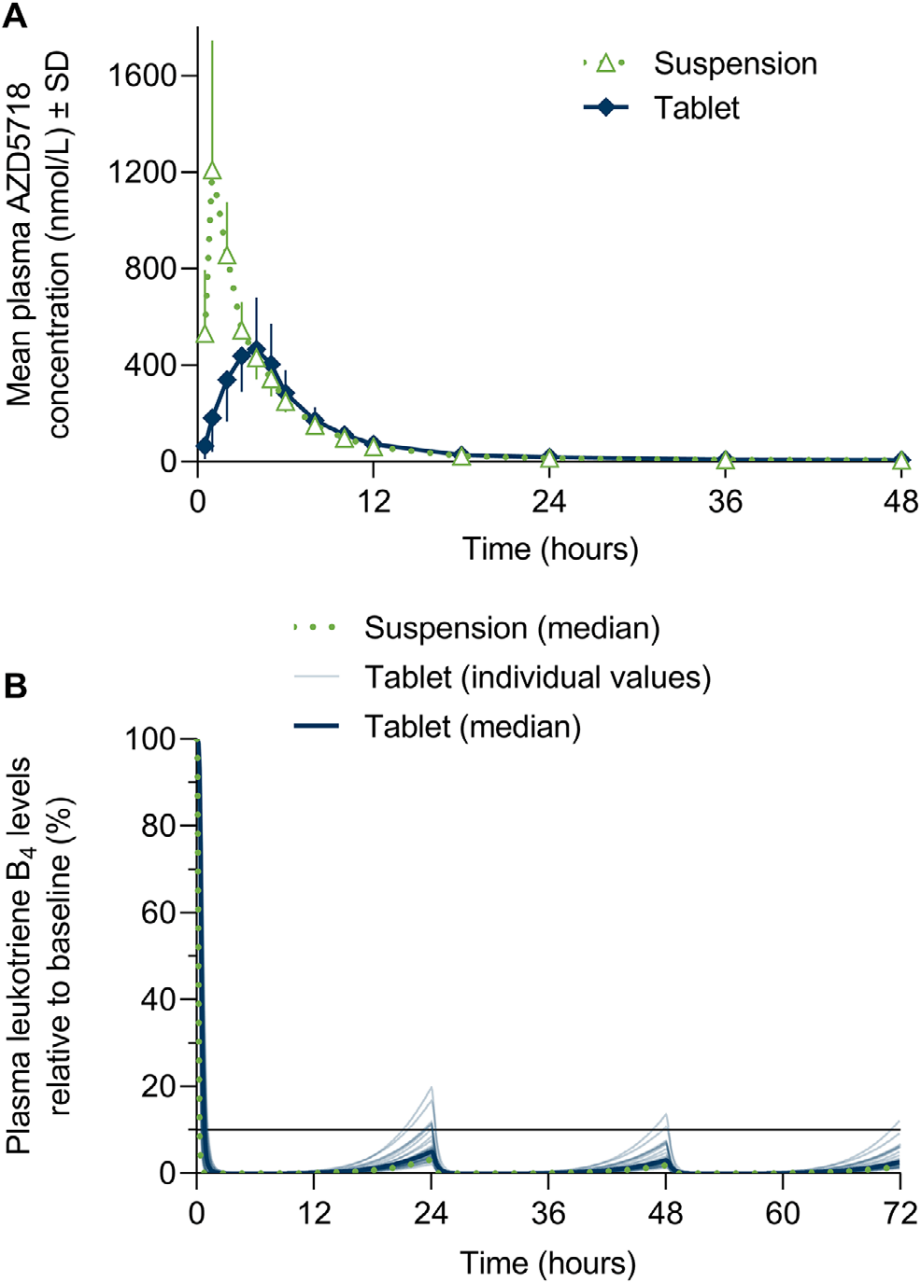
Single‐dose plasma concentration–time profile (A) and simulated multiple‐dose pharmacodynamics (B) of AZD5718 200 mg after administration of immediate‐release tablets or oral suspension. Horizontal line in (B) shows 90% inhibition of plasma leukotriene B_4_ levels. Molecular weight of AZD5718 is 446.5 g/mol.

Following a single oral doses of 200 mg, AZD5718 was absorbed more slowly when formulated as an immediate‐release tablet than as a suspension, with increased t_max_ and decreased C_max_ (Figure 3A; Table 3). Median t_max_ increased to 3.00 hours (range, 2.00‐5.00 hours) for the tablet formulation, compared with 1.00 hour (range, 0.98‐2.02 hours), and C_max_ decreased to 45% (90%CI, 36%‐57%) of the value for the suspension, expressed as a ratio of geometric LS means. Plasma concentrations of AZD5718 declined in an apparently biphasic manner, with very similar elimination half‐lives for the 2 formulations (Figure 3A; Table 3). Systemic exposure to AZD5718 was lower for the tablet formulation relative to the suspension, with a geometric LS mean AUC_0‐∞_ ratio of 72% (90%CI, 64%‐80%).

In multiple‐dose pharmacodynamic simulations, predicted plasma leukotriene B_4_ levels decreased to similar extents following once‐daily oral administration of AZD5718 formulated as tablets or suspension (Figure 3B). Simulated median leukotriene B_4_ levels remained below a target of 90% inhibition with both formulations, once steady state was reached (Figure 3B).

### AZD5718 Immediate‐Release Tablet Under Fed and Fasted Conditions

**Figure 4:**
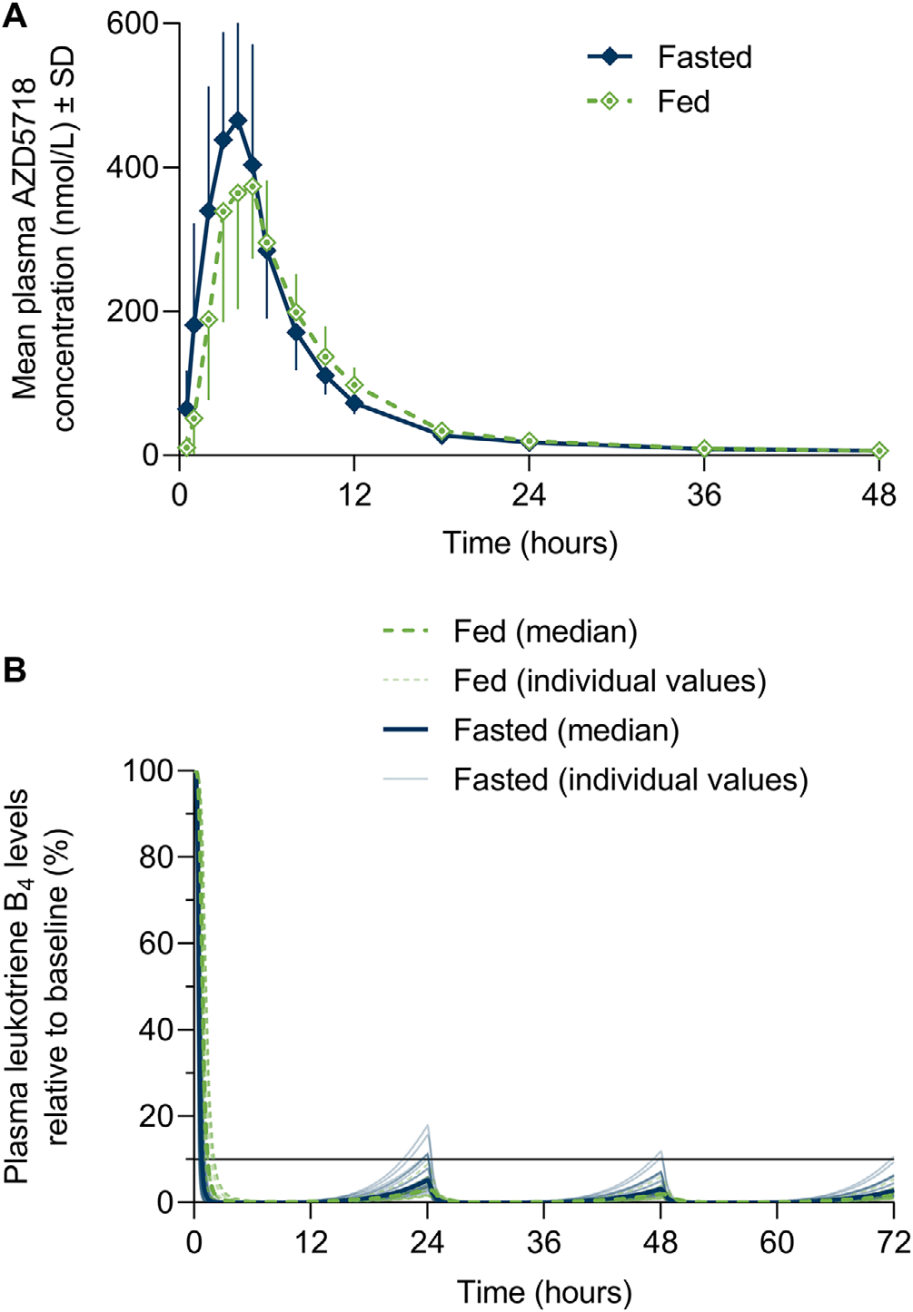
Single‐dose plasma concentration–time profile (A) and simulated multiple‐dose pharmacodynamics (B) of AZD5718 200‐mg immediate‐release tablets after administration in fasted and fed conditions. Horizontal line in (B) shows 90% inhibition of plasma leukotriene B_4_ levels. Molecular weight of AZD5718 is 446.5 g/mol.

Absorption of AZD5718 200 mg was delayed when tablets were taken after a high‐fat breakfast compared with overnight fasting (Figure 4A; Table 3). Median t_max_ increased to 4.00 hours (range, 3.00‐5.98 hours) in the fed state from 3.00 hours (range, 2.00‐5.00 hours) in the fasted state. C_max_ was reduced in the fed state, with a geometric LS mean ratio of 77% (90%CI, 63%‐95%) relative to the fasted state. Mean plasma elimination half‐life was not affected by food intake. Systemic exposure to AZD5718 was similar in the fed state relative to the fasted state, with a geometric LS mean AUC_0‐∞_ ratio of 96% (90%CI, 87%‐106%).

Variability in pharmacokinetic parameters between participants receiving AZD5718 tablets was low for AUC_0‐∞_ and AUC_0‐last_ (CV, <25%) and moderate for C_max_ (CV, 29%‐41%); see Table 3.

In multiple‐dose pharmacodynamic simulations, predicted leukotriene B_4_ levels remained below a target of 90% inhibition following administration of AZD5718 immediate‐release tablets once daily after food or fasting (Figure 4B).

### Safety and Tolerability

Six of the 12 participants reported a total of 11 adverse events (Table 4). No deaths, serious adverse events, or adverse events leading to discontinuation occurred. Headache was the only adverse event reported in more than 1 participant. All adverse events were of mild intensity except for poor‐quality sleep (1 participant) and influenza‐like illness (1 participant), both of moderate intensity. Five adverse events were considered possibly related to AZD5718: headache (1 participant), nausea (1 participant), nasal congestion (1 participant), oropharyngeal pain (1 participant), and hot flush (1 participant); all were of mild intensity.

**Table 4:** Participants Experiencing at Least 1 Adverse Event

		AZD5718 200 mg				
	Fasted				Fed	
Participants With	Rosuvastatin 10 mg (n = 12)	Tablet Plus Rosuvastatin 10 mg (n = 12)	Tablet (n = 12)	Suspension (n = 12)	Tablet (n = 12)	All (n = 12)
Any AE, n (%)	1 (8.3)	1 (8.3)	3 (25.0)	2 (16.7)	1 (8.3)	6 (50.0)
AEs						
Headache, n (%)	1 (8.3)	0	0	1 (8.3)^a^	0	2 (16.7)
Poor‐quality sleep, n (%)	0	0	1 (8.3)^b^	0	0	1 (8.3)
Nausea, n (%)	0	0	0	1 (8.3)^a^	0	1 (8.3)
Influenza‐like illness, n (%)	0	0	0	1 (8.3)^b^	0	1 (8.3)
Nasopharyngitis, n (%)	0	0	0	0	1 (8.3)	1 (8.3)
Musculoskeletal pain, n (%)	0	1 (8.3)	0	0	0	1 (8.3)
Nasal congestion, n (%)	0	0	1 (8.3)^a^	0	0	1 (8.3)
Oropharyngeal pain, n (%)	0	0	1 (8.3)^a^	0	0	1 (8.3)
Dry skin, n (%)	0	0	1 (8.3)	0	0	1 (8.3)
Hot flush, n (%)	0	0	0	1 (8.3)^a^	0	1 (8.3)

AE, adverse event.

AEs were coded using Medical Dictionary for Regulatory Activities (MedDRA) version 19.1 vocabulary.

a Considered possibly related to AZD5718 by the investigator (all other AEs were unrelated).

b Moderate intensity (all other AEs were mild).

No clinically relevant changes in participants’ vital signs, electrocardiography parameters, or clinical laboratory parameters were observed.

## Discussion

This phase 1 pharmacokinetic study of the FLAP inhibitor AZD5718 in healthy volunteers demonstrated that AZD5718 can be coadministered with statins. Furthermore, inhibition of leukotriene biosynthesis by AZD5718 was equivalent to immediate‐release tablets (given before or after food) and oral suspension in pharmacodynamic simulations. These findings support further clinical development of AZD5718 as a treatment for patients with coronary artery disease or other inflammatory cardiovascular diseases.

Patients with coronary artery disease receive high‐dose statin therapy to lower plasma lipid levels.^1^ Systemic exposure to rosuvastatin remained unchanged after coadministration with AZD5718 tablets, although rosuvastatin plasma t_max_ was reduced and C_max_ was increased by about 25%. Coadministration of AZD5718 with rosuvastatin had no effect on any safety outcome. In vitro, AZD5718 weakly inhibited the hepatic statin uptake transporter OATP1B1 (IC_50_ = 2.3 µM) and intestinal efflux transporter BCRP (IC_50_ = 20 µM; unpublished data); inhibition of the intestinal statin uptake transporter OATP2B1 was not studied. The findings of the present study do not allow us to conclude whether the observed drug‐drug interaction resulted from inhibition of intestinal transport via BCRP, inhibition of liver uptake via OATP1B1, or both. The interaction between rosuvastatin and the hepatic statin transporter inhibitor gemfibrozil indicates that OAT1B1 inhibition may be responsible. Rosuvastatin t_max_ was reduced and C_max_ was increased when coadministered with gemfibrozil, although the drug‐drug interaction was larger than for AZD5718, with 2‐fold increases in AUC and C_max_.^20^ The unchanged systemic exposure to rosuvastatin and the unchanged pharmacokinetics of AZD5718 after coadministration in the present study indicate that AZD5718 is likely to be fully compatible with high‐dose statin therapy in patients with coronary artery disease.

When taken as immediate‐release oral tablets, the bioavailability of AZD5718 was lower relative to the oral suspension used in the previous phase 1 study,^15^ with a decrease in systemic exposure of about 26%‐28% and a decrease in C_max_ of about 55%. The bioavailability of AZD5718 was unchanged when tablets were taken after a high‐fat breakfast relative to fasting conditions, although C_max_ was decreased by about 23% and t_max_ was delayed by about 1 hour. Because leukotriene B_4_ levels were not measured in this single‐dose study, we used a pharmacokinetic‐pharmacodynamic model to simulate the effect of multiple doses of AZD5718 on inhibition of leukotriene B_4_ biosynthesis. The observed minor pharmacokinetic differences did not translate to pharmacodynamic changes. Simulated pharmacodynamics were similar whether AZD5718 was taken as tablets or oral suspension and whether tablets were taken with or without food. Plasma leukotriene B_4_ levels after multiple daily doses of AZD5718 remained below a target of 90% inhibition at steady state. This indicates that AZD5718 concentrations are sufficient to inhibit leukotriene B_4_ biosynthesis to potentially therapeutic levels, regardless of the minor effects of formulation or food intake on its pharmacokinetic profile. These findings supported the use of immediate‐release tablets taken with no restriction on food intake in the ongoing phase 2a study of AZD5718 in patients with coronary artery disease (NCT03317002; Eva Prescott et al, in preparation).

AZD5718 was well tolerated and had a good safety profile in the present study as in the previous multiple‐dose first‐in‐human study.^15^ All participants completed the present study, including all 5 treatment periods. No deaths or serious adverse events occurred during the study, and none of the participants had an adverse event leading to discontinuation. No clinically concerning trends were noted in any of the assessed safety parameters.

A strength of this study was the use of a randomized crossover design to minimize the number of healthy volunteers exposed to the investigational new drug, while enabling the investigation of 3 questions relevant to further clinical development of AZD5718. Despite not measuring leukotriene B_4_ levels in the present study, we were able to evaluate leukotriene B_4_ inhibition after multiple doses of AZD5718 immediate‐release tablets using simulations based on a pharmacokinetic‐pharmacodynamic model developed with data from the first‐in‐human phase 1 study.^15^

## Conclusions

In this phase 1 study in healthy volunteers, AZD5718 oral suspension and immediate‐release tablets had different pharmacokinetic profiles, but were predicted in pharmacodynamic models to have the same inhibitory effects on production of proinflammatory leukotriene B_4_. The model predictions guided the dosing regimen for phase 2a development using convenient AZD5718 tablets taken once daily with or without food. Furthermore, the results of the present study allayed preclinical concerns over a potential drug‐drug interaction with statins by demonstrating that coadministration of AZD5718 with rosuvastatin had no clinically relevant effect on the safety and pharmacokinetic profiles of either drug. Together with the additional safety and tolerability data, these findings supported the design and initiation of an ongoing international phase 2a study of the efficacy and safety of AZD5718 in patients with coronary artery disease receiving the current standard of care.

## Conflicts of Interest

M.A. and P.F. are employees of Parexel. All other authors are employees of AstraZeneca, and may own stock or stock options. Sarah Sabir, PhD, and Matt Cottingham, DPhil, of Oxford PharmaGenesis provided medical writing support funded by AstraZeneca.

## Funding

This study was funded by AstraZeneca. AstraZeneca develops and markets treatments for cardiovascular disease. AZD5718 is an investigational medical product with no approved indication. Parexel received funding from AstraZeneca to conduct the study.

## Data Sharing

Data underlying the findings described in this article may be obtained in accordance with AstraZeneca's data‐sharing policy described at https://astrazenecagrouptrials.pharmacm.com/ST/Submission/Disclosure.

## References

[1] Task Force Members , Montalescot G , Sechtem U , Achenbach S , et al. 2013 ESC guidelines on the management of stable coronary artery disease: the Task Force on the management of stable coronary artery disease of the European Society of Cardiology. Eur Heart J. 2013;34(38):2949‐3003. 23996286 10.1093/eurheartj/eht296

[2] GBD 2013 Mortality and Causes of Death Collaborators . Global, regional, and national age‐sex specific all‐cause and cause‐specific mortality for 240 causes of death, 1990–2013: a systematic analysis for the Global Burden of Disease Study 2013. Lancet. 2015;385(9963):117‐171. 25530442 10.1016/S0140-6736(14)61682-2 PMC4340604

[3] Ridker PM , Lüscher TF . Anti‐inflammatory therapies for cardiovascular disease. Eur Heart J. 2014;35(27):1782‐1791. 24864079 10.1093/eurheartj/ehu203 PMC4155455

[4] Sabatine MS , Giugliano RP , Pedersen TR . Evolocumab in patients with cardiovascular disease. N Engl J Med. 2017;377(8):787‐788. 10.1056/NEJMc1708587 28834471

[5] Ridker PM , Everett BM , Thuren T , et al. Antiinflammatory therapy with canakinumab for atherosclerotic disease. N Engl J Med. 2017;377(12):1119‐1131. 28845751 10.1056/NEJMoa1707914

[6] Funk CD . Leukotriene modifiers as potential therapeutics for cardiovascular disease. Nat Rev Drug Discov. 2005;4(8):664‐672. 16041318 10.1038/nrd1796

[7] Carry M , Korley V , Willerson JT , Weigelt L , Ford‐Hutchinson AW , Tagari P . Increased urinary leukotriene excretion in patients with cardiac ischemia. In vivo evidence for 5‐lipoxygenase activation. Circulation. 1992;85(1):230‐236. 1309444 10.1161/01.cir.85.1.230

[8] Cipollone F , Mezzetti A , Fazia ML , et al. Association between 5‐lipoxygenase expression and plaque instability in humans. Arterioscler Thromb Vasc Biol. 2005;25(8):1665‐1670. 15933245 10.1161/01.ATV.0000172632.96987.2d

[9] Sanchez‐Galan E , Gomez‐Hernandez A , Vidal C , et al. Leukotriene B4 enhances the activity of nuclear factor‐kappaB pathway through BLT1 and BLT2 receptors in atherosclerosis. Cardiovasc Res. 2009;81(1):216‐225. 18852255 10.1093/cvr/cvn277

[10] Spanbroek R , Grabner R , Lötzer K , et al. Expanding expression of the 5‐lipoxygenase pathway within the arterial wall during human atherogenesis. Proc Natl Acad Sci U S A. 2003;100(3):1238‐1243. 12552108 10.1073/pnas.242716099 PMC298757

[11] Allen S , Dashwood M , Morrison K , Yacoub M . Differential leukotriene constrictor responses in human atherosclerotic coronary arteries. Circulation. 1998;97(24):2406‐2413. 9641692 10.1161/01.cir.97.24.2406

[12] Helgadottir A , Manolescu A , Thorleifsson G , et al. The gene encoding 5‐lipoxygenase activating protein confers risk of myocardial infarction and stroke. Nat Genet. 2004;36(3):233‐239. 14770184 10.1038/ng1311

[13] Tardif JC , L'Allier P L , Ibrahim R , et al. Treatment with 5‐lipoxygenase inhibitor VIA‐2291 (Atreleuton) in patients with recent acute coronary syndrome. Circ Cardiovasc Imaging. 2010;3(3):298‐307. 20190281 10.1161/CIRCIMAGING.110.937169

[14] Patel RSS , Hamid S , Blanco RR , et al. The 5‐lipoxygenase inhibitor zileuton improves endothelial function in carriers of coronary heart disease risk haplotypes in the ALOX5AP and LTA4H leukotriene pathway genes. Circulation. 2011;124(21_MeetingAbstracts) (Supplement 1).

[15] Ericsson H , Nelander K , Lagerstrom‐Fermer M , et al. Initial clinical experience with AZD5718, a novel once daily oral 5‐lipoxygenase activating protein inhibitor. Clin Transl Sci. 2018;11(3):330‐338. 29517132 10.1111/cts.12546 PMC5944575

[16] Pettersen D , Broddefalk J , Emtenas H , et al. Discovery and early clinical development of an inhibitor of 5‐lipoxygenase activating protein (AZD5718) for treatment of coronary artery disease. J Med Chem. 2019;62(9):4312‐4324. 30869888 10.1021/acs.jmedchem.8b02004

[17] Hua WJ , Hua WX , Fang HJ . The role of OATP1B1 and BCRP in pharmacokinetics and DDI of novel statins. Cardiovasc Ther. 2012;30(5):e234‐241. 21884024 10.1111/j.1755-5922.2011.00290.x

[18] Yusuf S , Bosch J , Dagenais G , et al. Cholesterol lowering in intermediate‐risk persons without cardiovascular disease. N Engl J Med. 2016;374(21):2021‐2031. 27040132 10.1056/NEJMoa1600176

[19] Martin P , Gillen M , Ritter J , et al. Effects of fostamatinib on the pharmacokinetics of oral contraceptive, warfarin, and the statins rosuvastatin and simvastatin: results from Phase I clinical studies. Drugs R D. 2016;16(1):93‐107. 26748647 10.1007/s40268-015-0120-x PMC4767723

[20] Schneck DW , Birmingham BK , Zalikowski JA , et al. The effect of gemfibrozil on the pharmacokinetics of rosuvastatin. Clin Pharmacol Ther. 2004;75(5):455‐463. 15116058 10.1016/j.clpt.2003.12.014

